# Vesicular Glutamate Release Is Necessary for Neural Tube Formation

**DOI:** 10.1523/JNEUROSCI.0681-25.2026

**Published:** 2026-02-18

**Authors:** Raman Goyal, Patricio A. Castro, Jacqueline B. Levin, Sangwoo Shim, Olga A. Balashova, Alexios A. Panoutsopoulos, Nicolas A. Fuentes, Grace Or Mizuno, Lin Tian, Laura N. Borodinsky

**Affiliations:** ^1^Department of Physiology & Membrane Biology, Shriners Hospitals for Children Northern California, University of California Davis, School of Medicine, Sacramento, California; ^2^Laboratory of Neural Development, LAND, Departamento de Fisiología, Facultad de Ciencias Biológicas, Universidad de Concepción, Concepción 4030000, Chile; ^3^Department of Biochemistry & Molecular Medicine, University of California Davis, School of Medicine, Sacramento, California

**Keywords:** calcium transients, glutamate release, neural stem cell proliferation, neural tube formation

## Abstract

The brain and spinal cord originate from a neural tube that is preceded by a flat structure known as the neural plate during early embryogenesis. In humans, failure of the neural plate to convert into a tube by the fourth week of pregnancy leads to neural tube defects (NTDs), birth defects with serious neurological consequences. The signaling mechanisms governing the process of neural tube morphogenesis are unclear. Here we show that in *Xenopus laevis* embryos, glutamate is released during neural plate folding in a Ca^2+^ and vesicular glutamate transporter-1 (VGluT1)-dependent manner. Vesicular release of glutamate elicits Ca^2+^ transients in neural plate cells that correlate with activation of Erk1/2. Knocking down or out VGluT1, globally or neural tissue-specifically, leads to NTDs and increased expression of Sox2, neural stem cell transcription factor, and neural plate cell proliferation. Exposure during early pregnancy to neuroactive drugs that disrupt these signaling mechanisms might increase the risk of NTDs in offspring.

## Significance Statement

Neural tube defects are serious and common birth defects that occur when the neural tube fails to form and close at 4 weeks of pregnancy. Use of antiepileptic drugs during early pregnancy increases the risk of these defects by unclear mechanisms. Here we show that vesicular release of glutamate occurs during and is necessary for neural tube formation in *Xenopus laevis* embryos. This study motivates discussion on the presynaptogenic signaling mechanisms in the nervous system and their role during these early developmental stages, challenging the prevailing paradigm that neurotransmission is not apparent until neurons fully differentiate and synapses are formed.

## Introduction

One of the first steps in nervous system development is the formation of the neural tube, which constitutes the presumptive brain and spinal cord, that originates from a flat layer of neuroectodermal stem cells called the neural plate. Failure in neural tube morphogenesis leads to birth defects known as neural tube defects (NTDs) that can be lethal or result in various neurological disabilities (Wallingford et al., 2013; Nikolopoulou et al., 2017).

The cellular events that are required for the morphogenesis of the neural tube include apical constriction of neural plate cells to enable bending, dorsoventral cell elongation to facilitate the curvature needed for the reshaping of the tissue, cell intercalation to enable the process of convergent extension, and cell migration toward the midline for fusion and closure of the neural tube (Ybot-Gonzalez and Copp, 1999; Ybot-Gonzalez et al., 2002; Haigo et al., 2003; Park et al., 2005; Lee and Harland, 2010; Ossipova et al., 2014; Nikolopoulou et al., 2017). In addition, the role of cell proliferation in neural tube formation has been a subject of extensive investigation (Jelínek and Friebová, 1966; Copp et al., 1988a; Harris and Hartenstein, 1991; Leise and Mueller, 2004; Ciruna et al., 2006; McShane et al., 2015; Parchem et al., 2015; Yang et al., 2015; Herrlinger et al., 2019; Pokrovsky et al., 2021). These studies show that increase or decrease in neuroepithelial cell proliferation can lead to NTDs in various species. Interestingly, in *Xenopus laevis* embryos, blocking cell proliferation following gastrulation does not interfere with neural tube closure (Harris and Hartenstein, 1991; Pokrovsky et al., 2021). In contrast, inhibiting cell cycle progression in embryos in which genetic (curly tail mouse; Copp et al., 1988b) or pharmacological (valproic acid-incubated *X. laevis* embryo; Sequerra et al., 2018) perturbations induced imbalanced neural plate cell proliferation, partially rescues the NTD phenotype. Overall, these studies suggest that precise regulation of cell proliferation is important for the morphogenesis of the neural tube.

Although several molecules that participate in these cellular events have been identified, the signaling mechanisms that operate in neural plate cells are unclear.

The causes of NTDs appear to be both genetic and environmental. Antiepileptic drugs are considered a risk factor for NTDs (Nakane et al., 1980; Lindhout et al., 1992; Omtzigt et al., 1992; Kaneko et al., 1999; Werler et al., 2011; Wlodarczyk et al., 2012). Although the prevailing view has been that these drugs interfere with neural tube morphogenesis through off-target effects (Eyal et al., 2004), our previous study has demonstrated that valproic acid, a commonly used antiepileptic drug, induces NTDs in frog embryos by a similar cellular mechanism as interfering with NMDA receptor-dependent glutamate signaling, which perturbs regulation of neural plate cell proliferation. Moreover, valproic acid-induced NTDs are partially rescued not just by inhibiting cell proliferation, but also by enhancing glutamate signaling (Sequerra et al., 2018). These studies suggest that the regulation of cell proliferation that is important for neural tube formation is dependent on glutamate signaling.

The mechanism by which glutamate is released in a developmental stage when neurons are not yet fully differentiated and synapses are not formed is unclear. Synaptic glutamate release is dependent on the expression and compartmentalization of key molecules that participate in the storing of glutamate in synaptic vesicles, anchoring of vesicles in synaptic terminals, fusion of vesicles, and release of neurotransmitter (Zhou and Danbolt, 2014). Prominently, vesicular glutamate transporters are indispensable for filling synaptic vesicles with glutamate in glutamatergic synapses (Hackett and Ueda, 2015). Whether aspects of the mechanism of vesicular glutamate release in synapses are also recruited at the early stages of neural plate folding has never been investigated before.

Here we show that the vesicular glutamate transporter 1 (VGluT1) is expressed in the neural plate and is necessary for neural tube formation. This study demonstrates VGluT1-dependent release of glutamate that mediates Ca^2+^ dynamics to regulate neural plate cell proliferation and morphogenesis of the neural tube.

## Materials and Methods

### Animals

All experimental procedures and research design utilized in this study complied with ethical regulations. The Institutional Animal Care and Use Committee approved the animal protocol #22264 implemented in this study. IACUC follows the guidelines established by the Animal Welfare Act and the Public Health Service Policy on Humane Care and Use of Laboratory Animals.

### *Xenopus laevis* animal handling and in vitro fertilization

Mature oocytes were collected in a dish from a previously hCG injected female frog and incubated with a small piece of minced testis. This is considered time 0 of fertilization. Fertilized oocytes were kept in 10% MMR saline, containing the following (in mM): 10 NaCl, 0.2 KCl, 0.1 MgSO_4_, 0.5 HEPES, 5 EDTA, and 0.2 CaCl_2_. Dejellying of embryos was done by briefly swirling fertilized eggs in 2% cysteine solution, pH 8. Developmental stages were recorded according to Nieuwkoop and Faber (Nieuwkoop and Faber, 1994). Animals were handled according to the IACUC guidelines using humane procedures to prevent animal suffering.

### Gene expression assays

Neural plates from nine mid-neural plate stage (stage 16) *X. laevis* embryos were isolated by incubating dorsal halves of embryos with 1 mg/ml collagenase for 1 min. This allows to dissect out the neural plate from mesodermal and endodermal tissues. Dissected neural plates were then resuspended in TRIzol reagent (Invitrogen, catalog #15596026) and stored at −80°C. RNA was extracted with kit according to manufacturer's instructions (RNeasy Mini Kit, Qiagen, catalog #74104), gDNA was eliminated (RapidOut DNA Removal Kit, Thermo Scientific, catalog #00859896), and cDNA was made (High-Capacity cDNA Reverse Transcription Kit, Applied Biosystems, catalog #00890068) with standard protocols. Using this cDNA as template, RT-PCR was performed with the following primers: vesicular glutamate transporter 1 (*vglut1*): forward, GCAACTTGGGTGTAGCCATT, reverse, TGCCCATTTACTCCAGATCC; excitatory amino acid transporter 5 (*eaat5*): forward, GTGGGATGTCTGCTTGGATT, reverse, ATGTGGCTTCCACAAGGTTC; syntaxin 1A (*stx1a*): forward, ATGAAGGATCGGACCAGGGA, reverse, TGTGGCGTTGTATTCGGACA; vesicle-associated membrane protein 1 (*vamp1*): forward, GCCACAGGTGATCCTGGAAA, reverse, AGGAGACGCTCCACACAATG; synaptosome-associated protein 25 (*snap25*) forward, AAGGCTTGGGGCAATAACCA, reverse, AACCACTGCCCAGCATCTTT, designed using Primer-BLAST (www.ncbi.nlm.nih.gov). All sequences are written from 5′ to 3′.

Quantitative RT-PCR for assessment of developmental *vglut1* expression and comparison of *vglut1*, *vglut2*, and *vglut3* was performed by homogenizing embryos in TRIzol reagent (Invitrogen, catalog #15596026) and storing samples at −80°C. RNA was extracted with kit according to the manufacturer's instructions (RNeasy Mini Kit, Qiagen, catalog #74104), gDNA was eliminated (RapidOut DNA Removal Kit, Thermo Scientific, catalog #00859896), and cDNA was made (High Capacity cDNA Reverse Transcription Kit, Applied Biosystems, catalog #00890068) with standard protocols. Using cDNA as template, qRT-PCR was performed with SYBR Green Universal Master Mix (Applied Biosystems, catalog #2107118) in the Stratagene Mx3005 real-time PCR machine. RT-PCR program: 15 min at 95°C, 50 cycles of 45 s at 95°C/30 s at 47°C/30 s at 72°C, 1 min at 95°C, 30 s at 47°C, and 30 s at 95°C. Primers used were as follows: *vglut1*, forward: CCACGGGATCTGGAGTAAATG, reverse: CTGAACTCCACCCAGAGTATTG; or forward: TACACTAGGACGTCTGCACAGGAT, reverse: CGAGGAAGCCCAAAGCAAGTACAA; *odc*, forward: GTCAATGATGGAGTGTATGGATC, reverse: TCCATTCCGCTCTCCTGAGCAC; *vglut2*, forward: GCAGGAGCTGTTATTGCTATGCCA, reverse: GAAGAGGTACCAAACCAGGCCAAA; *vglut3*, forward: TCAGACTCAGCCACCTCCAAGATT, reverse: ATGCAAAACCCCAAGCCACTCA; *sub1*, forward: CAACTGAAGGAGCAGATGTCGGAT, reverse: CATGGTTTCGTCAAGGCGTAGGTA. All sequences are written from 5′ to 3′.

### VGluT1 knockdown

Two-cell stage embryos were unilaterally or bilaterally injected with 2–8 pmol of translation blocking morpholino (MO) VGluT1-MO1 TCCTAAACTCCATTGTGATCCTCCT (VGluT1-KD1/KD) or with 8 pmol splicing-blocking MO (VGluT1-KD2) VGluT1-MO2 ATGTTTTTTCCTTACCTCGATAACA per blastomere (Gene Tools). Controls were sibling embryos injected with standard control MO, CCTCTTACCTCAGTTACAATTTATA (Control). Morpholinos were injected along with dextran-Alexa Fluor conjugates or with GFP or mCherry mRNA to assure permanency of MO reporter after PFA or TCA fixation. All sequences are written 5′ to 3′. Rescue experiments were implemented by injecting 250 pg of VGluT1-MO-resistant *X. laevis vglut1*-mRNA (125 pg/blastomere) along with VGluT1-MO. *X. laevis* VGluT1 mRNA was modified by substitution of 5’ UTR with a fragment of the Kozak consensus sequence gcc acc (Kozak, 1987). A number of wobble mutations were introduced in the VGluT1 coding region to prevent VGluT1-MO binding to modified *vglut1* mRNA. mRNA was synthesized as previously described (Borodinsky et al., 2004; Belgacem and Borodinsky, 2011; Swapna and Borodinsky, 2012; Balashova et al., 2017). Assessment of VGluT1-KD efficiency was performed by Western blot and PCR assays (splicing-blocking MO).

Assessment of VGlut1 splicing-blocking MO (MO2) efficiency was performed by homogenizing 9 stage 15 embryos in each group in TRIzol reagent (Invitrogen, catalog #15596026). Samples were stored at −80°C. RNA was extracted with kit according to manufacturer's instructions (RNeasy Mini Kit, Qiagen, catalog #74104), gDNA was eliminated (RapidOut DNA Removal Kit, Thermo Scientific, catalog #00859896), and cDNA was made (High-Capacity cDNA Reverse Transcription Kit, Applied Biosystems, catalog #00890068) with standard protocols. Using this cDNA as template, PCR was performed for Control-MO and VGluT1-MO2 injected embryos with primers located in exon 1 and exon 2 that generates no PCR product from cDNA of non-spliced *vglut1* transcript and a 100 bp PCR product when the mRNA is spliced correctly (MO control). Forward primer: GCATGGTCAACAACAACACG, Reverse primer: GAACCGTGTATCATGCCGAC, written from 5′ to 3′.

### Western blot assays

To determine VGluT1 and Sox2 protein levels, neural tube, stage-22 whole embryos were homogenized in extraction buffer containing 1% Triton X-100, 150 mM NaCl, 25 mM HEPES, pH 7.4, 2 mM EDTA, and protease inhibitors cocktail (Thermo Fisher Scientific). Samples were centrifuged at 16,100 × *g* for 10 min and the pellet discarded. Supernatant was then boiled with Laemmli buffer and run on a 10% SDS-PAGE followed by protein transfer to PVDF membrane. PVDF membranes were probed with monoclonal anti-*Xenopus*-VGluT1 1:500 (GenScript, costume-made) in 5% milk, Sox2 1:500 (catalog #AF2018, R&D Systems) in 5% BSA followed by incubation with HRP-conjugated secondary antibodies 1:10,000 (Jackson ImmunoResearch) and visualized by Western Lightning Plus-ECL, Enhanced Chemiluminescence Substrate (catalog #NEL103E001, PerkinElmer). PVDF membranes were stripped in 0.2 M glycine HCl buffer, pH 2.5, 0.05% Tween for 20 min and reprobed with GAPDH 1:50,000 in 5% milk (catalog #60004-1-1g, Proteintech), as loading control, followed by incubation with HRP-conjugated secondary antibodies 1:20,000 (Jackson ImmunoResearch) and visualized by Western Lightning ECL (Millipore/Sigma, catalog #GERPN2106).

### Whole-mount immunostaining

Embryos at stage 13 through 18 were fixed in 4% PFA for 2 h at 23°C or overnight at 4°C and the dorsal half containing the neural plate was isolated and bleached in 1:2 Dent's fixative/H_2_O_2_ overnight at 23°C. Samples were washed, permeabilized in 1% Triton X-100, and incubated overnight at 4°C with primary antibodies, followed by staining with fluorescent secondary antibodies at 23°C for 2 h, and finally clearing overnight in benzyl benzoate. *Z*-stack confocal images of embryos or neural tissue (100 μm thick) were taken with a confocal microscope (Nikon C1 or C2), 10× or 20× objective, through ∼30–100 steps (3–10 μm) longitudinally through a dorsoventral direction. Primary antibodies used for whole-mount immunostaining were as follows: VGluT1 1:200 (catalog #AB5905, EMD-Millipore), Sox2 1:200 (catalog #AF2018, R&D Systems), pERK1/2 1:200 (catalog #4377S, Cell Signaling Technology), PCNA 1:1,000 (catalog #2586S, Cell Signaling Technology), PHH3 1:500 (catalog #06-570, EMD-Millipore), and GFP 1:500 (catalog #ab13970 Abcam).

Quantitative assessment of the number of cells immunopositive for VGluT1, Sox2, PCNA, and PHH3 was performed by using the Imaris “Spot” function to detect nuclei objects filtered by object size, fluorescence intensity, and the built-in quality threshold.

### Immunohistochemistry of tissue sections

Neural plate stage embryos were fixed at 23°C with 4% PFA for 10 min and processed for immunostaining as previously described (Balashova et al., 2017). Briefly, samples were paraffin-embedded, and 10-μm-thick transverse sections of the neural plate were incubated with primary and secondary antibodies overnight at 4°C and for 2 h at 23°C, respectively. Primary antibodies used were as follows: VGluT1/2 (SYSY, 1:1,000), β-tubulin (E7, 1:100, DSHB), and Myc-tag 1:2,000 (catalog #2276, Cell Signaling). Antigen retrieval was performed by boiling samples in 0.05% citraconic anhydride (Namimatsu et al., 2005) or in Diva antigen retrieval solution (Biomedical Care), pH 7.4, for 10 min in water bath. Samples were permeabilized with PBST (0.5% Triton) for 1 h at 23°C, blocked with 5% BSA in PBST (0.1% Triton) for 30 min using SNAP i.d. 2.0 System for immunohistochemistry (Millipore). Samples were imaged with a confocal microscope (Nikon A1), 60× objective through ∼15 1 μm steps.

### Electron microscopy

#### Transmitted EM

Embryos were fixed in 3% glutaraldehyde in 100 mM HEPES, pH 7.4, overnight at 4°C and washed several times in 100 mM HEPES and processed for routine TEM. Specimens were postfixed in 1% OsO_4_ in PBS overnight on ice and washed in PBS and water. Fixed embryos were embedded in epoxy blocks. Ultrathin sections were stained with uranyl acetate and lead citrate prior to viewing on a transmission electron microscope (Philips CM120 Biotwin Lens, FEI Company) using a Gatan MegaScan digital camera (model 794/20, 2K × 2K, Gatan).

#### Immuno-TEM

Embryos were fixed in 4% paraformaldehyde in 0.1 M sodium phosphate buffer at 4°C overnight. Ultrathin sections were incubated with primary and secondary antibodies overnight at 4°C and for 2 h at 23°C, respectively. Primary antibody was VGluT1 1:100–1:10,000 (catalog #AB5905, EMD-Millipore). Secondary antibody was gold particle (10 nm)-conjugated goat-anti-guinea pig IgG, EM grade, 1:40 (catalog #25329, Electron Microscopy Sciences). Immunolabeled sections were imaged on the same electron microscope as indicated before. Control samples consisted in embryos and ultrathin sections subjected to the same procedure except omission of primary antibody.

### Measurement of glutamate release

#### iGluSnFR

Two-cell stage wild type and unilaterally injected with 2 pmol VGluT1-MO1 or Control-MO + Alexa 647-dextran embryos were bilaterally injected with 8 ng iGluSnFR mRNA per embryo [iGluSnFR, a modified (pcDNA-spacer-123D5, L.T.'s Lab) derived from construct gift from Loren Looger, Addgene plasmid #41732 (Marvin et al., 2013)] that was previously subcloned into the pCS2^+^ vector. iGluSnFR-expressing stage 13–16 embryos were confocally time-lapse imaged at 1 Hz acquisition rate. Images were taken at different neural plate stages using Nikon confocal microscope in response to 2–5 μM ionomycin or DMSO for 5 min followed by 5 mM glutamate. Membrane-mCherry-expressing embryos were used as controls. Fluorescence intensity was measured using NIS Elements software.

#### Fluorometric assay

Neural plate stage (stage 15–15.5) wild type and morpholino-injected embryos were incubated with 100 nM tetanus toxin or DMSO for 1 h followed by 1 h incubation with 2 μM ionomycin in 200 μl volume. Bathing solution was collected and released glutamate measured with a Fluorometric Glutamate Assay Kit (STA-674 Cell Biolabs).

### In vivo Ca^2+^ imaging

DNA encoding the Ca^2+^ sensor GCaMP6s (pGP-CMV-GCaMP6s, a gift from Douglas Kim, HHMI Janelia Research Campus, Ashburn, Virginia; plasmid #40753, Addgene; Chen et al., 2013) was subcloned into the pCS2^+^ vector using BglII and NotI restriction sites. The BglII restriction site was included in pCS2^+^ with the following primers: forward, 5′-TCACTAAAGGGAACAAAAGATCTGGGTACCGGGCCCAA-3′; reverse, 5′-TTGGGCCCGGTACCCAGATCTTTTGTTCCCTTTAGTGA-3′. For all experiments in this study, mRNA was transcribed from the indicated plasmids using mMessage mMachine kits (Ambion). GCaMP6s mRNA was injected in two-cell stage embryos (1 ng mRNA/embryo). Neural plate stage embryos [14–19 h postfertilization (hpf)] were imaged under a confocal microscope at an acquisition rate of 0.05–0.1 Hz for 5 min to 2 h. Detection of Ca^2+^ transients was thresholded by a peak change in fluorescence of at least two times the noise, as in previous studies (Borodinsky et al., 2004; Belgacem and Borodinsky, 2011; Swapna and Borodinsky, 2012). The number of Ca^2+^ transients in unilaterally VGluT1-KD1 or Control embryos were compared, and significance was assessed by paired *t* test.

### Imaging of Erk1/2 activity

piRFP670 (a gift from Vladislav Verkhusha; Addgene plasmid #45457; Shcherbakova and Verkhusha, 2013) was subcloned into pCS2^+^ using KpnI and NotI (New England Biolabs; NEB). The ERK-sensitive region of ERKKTRClover from pENTRA-ERKKTRClover (a gift from Markus Covert; Addgene plasmid # 59138; Regot et al., 2014) was removed and amplified by PCR using the following primers: forward: CAAAGGTACCGGCAACATGGCAAAGGGCCGAAAGCC, reverse: CACCATACCGGTGAGGATGGGAATTG. These primers add KpnI and AgeI restriction sites, keep the iRFP in frame with the KTR, and optimize the Kozak sequence of the new construct for *X. laevis* expression (Kozak, 1991; Nakagawa et al., 2007). The PCR product and piRFP670-pCS2^+^ were both digested with KpnI and AgeI (NEB), gel purified, and ligated. The new plasmid was confirmed by restriction digests and RNA was made from the T3 promoter using Ambion's mMessage mMachine kit. The sensitivity of the new construct was confirmed in culture as described below using phorbol 12-myristate 13-acetate (PMA; agonist) or PD0325901 (antagonist).

Embryos were injected at the two-cell stage with a mixture of mRNA encoding GCaMP6s (1.5 ng), ERK-KTR-iRFP670 (1.5 ng), and H2B-mRFP1 (200 pg; mRNA made from the SP6 promoter of pCS2^+^-H2B-mRFP1, a gift from Sean Megason; Addgene plasmid # 53745), or 2 pmol VGluT1-MO or Control-MO (unilateral; along with tracer), 1.5 ng ERK-KTR-iRFP670 (bilateral), and 200 pg H2B-mRFP1 (bilateral). They were grown at 18–21°C until early neural plate stages when they were confocally imaged in the three channels.

For simultaneous Ca^2+^ and Erk1/2 activity analysis, nuclei were tracked by H2B signal in NIS Elements software (Nikon), and the intensity of all three channels in this H2B-RFP fluorescently labeled area (nuclear) and surrounding area (cytosolic) was measured over time. Cells were categorized into Ca^2+^-active or Ca^2+^-silent based on occurrence of Ca^2+^ transients or not during the recording, respectively. At least five cells of each category were analyzed per embryo (total: 70 cells). Ca^2+^ active cells were first selected and Ca^2+^-silent cells were sampled to match anteroposterior and mediolateral contralateral localization to those active in each embryo. Average of cytosolic/nuclear ERK-KTR-iRFP670 was calculated per group per embryo, and comparisons were performed with paired *t* test.

For assessing Erk1/2 activity dependence on VGluT1 expression, we imaged embryos at mid-neural plate stages (stage 15–16) and measured ERK-KTR-iRFP670 cytosolic/nuclear signal in cells of VGluT1-MO/Control-MO injected and wild type halves of neural plate. Cells were sampled to match anteroposterior and mediolateral localization across groups. Average of cytosolic/nuclear ERK-KTR-iRFP670 of at least five cells in each half of the neural plate from at least four embryos (total neural plate cells at least 40) was calculated per wild type and injected neural plate per embryo and comparisons were performed with paired *t* test.

### VGluT1 knock-out

Synthetic guidance RNA (sgRNA) targeting *vglut1* was designed using the CRISPRscan website (Moreno-Mateos et al., 2015) and inDelphi model (Shen et al., 2018), which provide in silico predictions for mutational outcomes. The VGluT1 sgRNA (guidance sequence: AGCCTGCTACGCTCCAGAGG) was synthesized using the EnGen sgRNA synthesis kit (New England Biolabs). The sgRNA was complexed with Cas9 protein (PNA Bio, #CP02) at 300 mM KCl to form ribonucleoprotein complexes and injected into embryos. The CrispantCal web tool (Burger et al., 2016) was used to calculate volumes corresponding to an optimal one-to-one molecular ratio of gRNA to Cas9 in a CRISPR-Cas9 injection mix. To quantify the editing efficiency, genomic DNA was extracted from five edited embryos at early neural plate stages (stage 14) using DNeasy Blood & Tissue Kit (Qiagen). The edited locus of *vglut1.s* was amplified from genomic DNA using primers, forward, 5′-TTTGCGCTTGACCCAGGTAT-3′; reverse, 5′-ACGGGCGACAATTTTATGCG-3′, specific to the CRISPR/Cas9-targeted site for Sanger sequencing. The sequencing results were used for in silico analysis of the INDELs generated by the CRISPR/Cas9-mediated editing using Inference of CRISPR Edit analysis software (Synthego).

### Neuroectoderm-specific VGluT1 knockdown

Eight-cell stage embryos were bilaterally injected with 2 pmol of translation blocking morpholino (MO) VGluT1-MO1 TCCTAAACTCCATTGTGATCCTCCT (VGluT1-KD1/KD) per dorsal blastomere (Gene Tools). Controls were sibling embryos injected in dorsal blastomeres with 2 pmol standard control MO, CCTCTTACCTCAGTTACAATTTATA (Control). Morpholinos were injected along with dextran-Alexa Fluor conjugates. All sequences are written 5′ to 3′.

### Assessment of neural tube defect phenotype

VGluT1-KD, VGluT1-KO, Control, and vglut1-mRNA injected embryos were imaged with a stereoscope when control embryos reached early neural tube stages (stage 20). Observed phenotypes were categorized in “Closed” or “Open” (“NTDs”) neural tube.

### Experimental design and statistical analyses

Rigorous research design and analysis were implemented by running all the controls necessary alongside experimental samples. Data analysis was performed blindly by the experimenter or through unbiased automation. Number of samples for each experiment was determined by power analysis of pilot experiments and replicated at least three times with at least five samples. No data were excluded from the analysis. Statistical analysis of the data was done with Prism software (GraphPad). Normality test was performed in each set of data and then parametric (normally distributed) or nonparametric statistical analysis was chosen. Paired tests were implemented in unilaterally manipulated embryos (comparison of noninjected and microinjected halves of neural tissue) or when Ca^2+^ activity or released glutamate (iGluSnFR) was compared before and after addition of an agent in the same sample. Differences were considered statistically different when *p* < 0.05. Type of statistical test used is indicated in the figure legends.

## Results

### VGluT1 is expressed in neural plate stage embryos and mediates glutamate release

**Figure 1.**
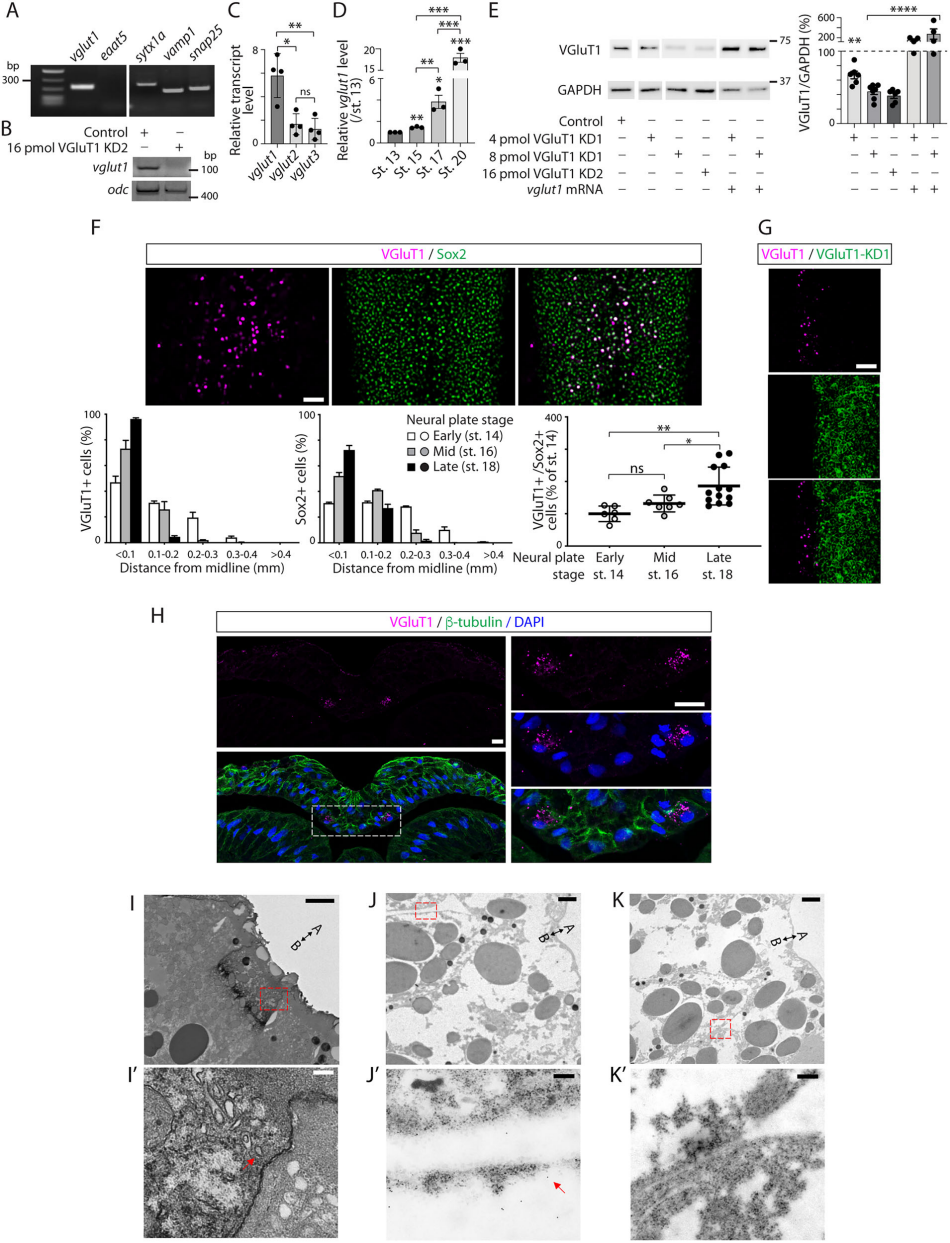
VGluT1 is expressed in the neural plate of *X. laevis* embryos. ***A–D***, RNA was isolated from embryos at mid-neural plate stages and reverse transcriptase (RT)-PCR assays were performed for *vglut1*, *eeat5*, *syntaxin1a* (*stx1a*), *vamp1*, and *snap25*. ***B***, Embryos were injected with a splicing-blocking morpholino targeting *vglut1* (VGluT1-KD2) or control morpholino (Control). RT-PCR was performed with primers flanking a sequence between exon 1 and 2 that can only be detected in correctly spliced transcript (control). Shown is representative example of RT-PCR product in control and VGluT1-KD2 samples, *N* = 3. ***C***, ***D***, Quantitative RT-PCR was performed from samples of embryos at stage 20 to compare relative expression of *vglut1*, *vglut2*, and *vglut3* (***C***) or with embryos at stages (st.) 13, 15, 17, and 20 to compare developmental regulation of *vglut1* during neural plate folding (***D***). Graphs show individual and mean ± SEM transcript level as ratio of control transcript (sub-1, ***C***) or as percent of *vglut1* transcript level in the youngest stage tested (st. 13, ***D***). *N* = 3 (***C***) and 4 (***D***) experiments, *p < 0.05, **p < 0.01, ***p < 0.001, one-way ANOVA (***C*** and ***D***), and one-sample *t* and Wilcoxon test [***D***, compared with hypothetical value of 1 (st. 13)]. ***E***, Western blot assays were performed in whole-cell lysates from neural-plate stage control, VGluT1-knockdown (KD), and *vglut1* mRNA-injected embryos. GAPDH was used as loading normalizer. Shown is a representative example. Graph shows normalized signal intensities from individual samples and means as percentages of control values (dashed line, 100%) from *N* ≥ 4 experiments. Statistical analysis was done with one-sample *t* and Wilcoxon test, **p < 0.01, ****p < 0.0001. ***F***, Representative maximum intensity projection of whole-mount immunostained dorsal half of mid-neural plate stage (st. 16) embryo for VGluT1 and Sox2 (neural stem cell marker). Graphs show mediolateral (mean percent of VGluT1+ (left) and Sox2+ (middle) cells per 100 μm bin from the midline compared with the total number of VGluT1+ and Sox2+ cells, respectively ± SEM) or developmental (right; individual data and mean percent of VGluT1+ cells of the total number of Sox2+ cells ± SEM) distribution of number of VGluT1 immunopositive (+) cells. **p < 0.01, ns, not significant, one-way ANOVA. *N* = 3. ***G***, VGluT1-KD decreases the number of VGluT1+ cells in the neural plate. Shown is a maximum intensity projection of whole-mount immunostained unilaterally VGluT1-KD neural plate from a representative embryo, *N* = 3. ***H***, Representative maximum intensity projection of 10-μm-thick transverse section of neural plate stage (st. 16) embryo for VGluT1 and β-tubulin, nuclei labeled with DAPI. Scale bars, 20 μm. ***I–K***, Neural plate stage (st. 18) embryos were processed for transmitted (***I***) and immuno-transmitted (***J***, ***K***) electron microscopy assays. Shown are representative examples. A, apical; B, basal. Dashed boxes in ***I–K*** indicate fields of view shown in ***I*’*–K*’**. Arrow in ***I*’** points to vesicular structures and in ***J*’** to structure with lipidic-looking background (vesicular) close to the cell–cell border immunopositive for VGluT1. ***J***, ***K***, Ultrathin sections were incubated with ***J***, ***J*’** or without ***K***, ***K*’** 1:3,000 VGluT1 antibody, followed by the same immunogold labeling procedure as indicated in Materials and Methods. *N* = 3, scale bars, 2 (***I–K***) and 0.2 (***I*’*–K*’**) μm.

To investigate the mechanisms of glutamate release in neural plate stages, we examined expression of VGluT1, the vesicular glutamate transporter known to be expressed in *X. laevis* (Borodinsky et al., 2004; Root et al., 2008; Session et al., 2016). mRNA expression and Western blot assays show that VGluT1 is expressed in neural plate stages (Fig. 1*A–E*), in agreement with previous studies (Root et al., 2008), while transcript for the excitatory amino acid transporter 5 (*eaat5*), known to be a retina-specific glutamate transporter (Arriza et al., 1997), is not detected during neural plate stages (Fig. 1*A*). *vglut1* transcript is the most abundant compared with *vglut2* and *3* (Fig. 1*C*), and its expression is upregulated with the progression of neural plate folding (Fig. 1*D*). VGluT1 protein is enriched in neural plate cells (Fig. 1*F*,*H*) and, similar to transcript levels, the number of VGluT1+/Sox2+ neural plate cells increases as neural plate folding progresses (Fig. 1*F*). The decreased transcript (Fig. 1*B*) and protein (Fig. 1*E*,*G*) levels when knocking down VGluT1 expression prove the specificity of immunodetection and efficiency of the knockdown approaches used. In addition, transcripts for other proteins associated with neurotransmitter vesicular release, such as syntaxin 1A (*stx1a*), vesicle-associated membrane protein 1 (*vamp1*), and synaptosome-associated protein 25 (*snap25*), are also detected at these neural plate stages (Fig. 1*A*), suggesting that the molecular machinery for vesicular glutamate release is available during neural tube formation. Indeed, ultrastructural assays we performed at these stages show that vesicular structures are prominent in neural plate cells (Fig. 1*I*,*I*’) and that VGluT1 is found in these subcellular compartments (Fig. 1*J*,*J*’), while control samples, in which VGluT1 antibody was omitted, show no immunogold particles (Fig. 1*K*,*K*’). This agrees with the pattern of fluorescent immunostaining in neural plate stage embryo transverse sections, where vesicular structures in close proximity to the nucleus of distinct neural plate cells are immunopositive for VGluT1 (Fig. 1*H*).

**Figure 2.**
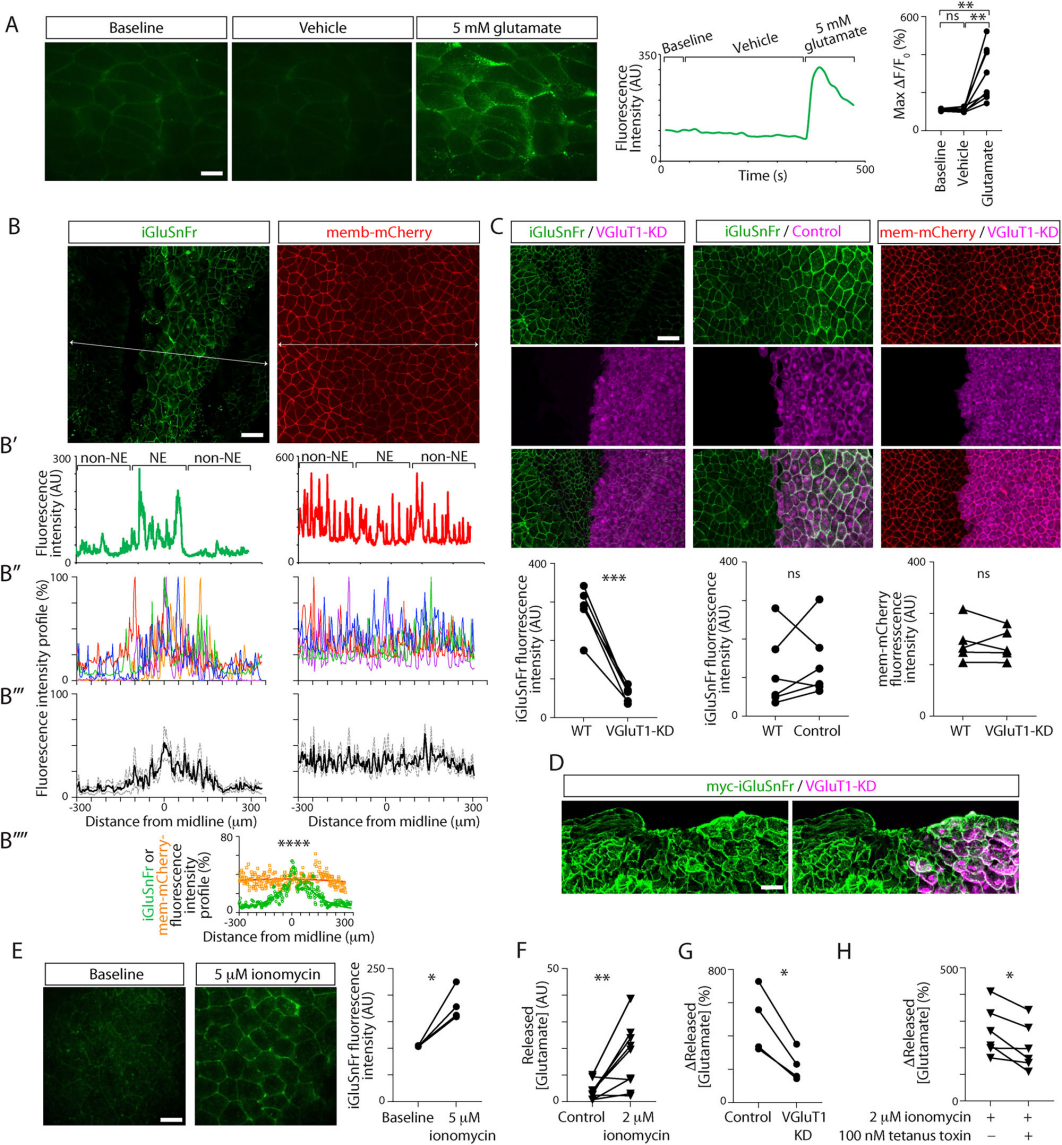
Glutamate is released from neural plate stage embryos in a VGluT1- and Ca^2+^-dependent manner. ***A–E***, Two-cell stage embryos were bilaterally microinjected with mRNA encoding iGluSnFR (***A–E***) or membrane (memb)-mCherry (***B***, ***C***) and unilaterally injected with VGluT1-morpholino 1 [VGluT1-knockdown (KD), ***C***, ***D***] or control-morpholino (Control, ***C***). Embryos were live imaged (***A–C***, ***E***) or processed for immunostaining (***D***). ***A***, iGluSnFR expressed in neural plate stage embryos senses extracellular glutamate levels. Shown are single time frames of time-lapse recording of the neural plate in whole embryo when either vehicle or 5 mM glutamate was added to the bathing solution. Trace shows representative change in iGluSnFR fluorescence intensity over time. Graph shows individual maximal change in iGluSnFR fluorescence intensity after addition of vehicle or 5 mM glutamate. *N* = 7, **p < 0.01, ns, not significant, paired ANOVA. ***B***, Released glutamate is higher in the neural plate. Shown is maximum intensity projection of confocal image of iGluSnFR- or memb-mCherry-expressing embryos and mediolateral fluorescence intensity profile for both reporters. While iGluSnFR exhibits higher intensity in membrane of neural plate cells located within 200 μm of the midline compared with non-neural ectodermal cells, memb-mCherry does not show a differential mediolateral distribution. Graphs show example (***B*’**), all individual experiments (***B*”**, ***B*’’’’**) and mean + SD (***B*’’’**) mediolateral iGluSnFR and mem-mCherry fluorescence intensity (***B*’**) and percent intensity profile (***B*’’*–B*’’’’**), *N* = 5 iGluSnFR- and *N* = 4 mem-mCherry-expressing embryos. Comparison of best fit curves for iGluSnFR and mem-mCherry shows significantly different fluorescence intensity profiles [***B*’’’’**, ****p < 0.0001, nonlinear regression fit, Lorentzian (Cauchy)]. ***C***, VGluT1-KD impairs glutamate release. Images are representative examples of maximum intensity projection of unilaterally manipulated embryos as indicated. Graphs represent iGluSnFR (left and middle) or memb-mCherry (right) fluorescence intensity of individual embryos in WT and manipulated halves of neural plate. ***p < 0.001, ns, not significant, *N* = 5 (VGluT1-KD iGluSnFR and memb-mCherry), *N* = 6 (Control), 2-tail paired *t* test. ***D***, Expression of iGluSnFR is not affected by VGluT1-KD. Shown is a representative example of 10 μm section of unilaterally VGluT1-KD neural plate immunostained for myc-tag linked to iGluSnFR construct, *N* = 3. ***E***, Ca^2+^ entry in neural plate cells increases glutamate release. Images show iGluSnFR-expressing neural plate before and after addition of 5 μM ionomycin. Graph shows individual data of % maximal change in iGluSnFR fluorescence intensity after addition of ionomycin. *N* = 4, *p < 0.05, one-sample *t* and Wilcoxon test, compared with the hypothetical value of 100 (before addition of ionomycin). ***F–H***, Neural plate stage wild type (***F***, ***H***) and bilaterally morpholino-injected embryos (***G***, Control and VGluT1-KD) were incubated with 2 μM ionomycin (***F–H***) or vehicle (Control, ***F***) in the presence (***H***) or absence (***F–H***) of 100 nM tetanus toxin for 20 min. Bathing solution was collected and released glutamate measured with a Fluorometric Glutamate Assay Kit. ***F***, Graph shows released glutamate concentration in Control (vehicle) and ionomycin treated embryos, *N* = 9, **p < 0.01, 2-tail paired *t* test. ***G***, Graph shows individual % change in released glutamate concentration in Control and VGluT1-KD embryos treated with ionomycin and compared with vehicle-treated (100%), *N* = 4, *p < 0.05, 2-tail paired *t* test. ***H***, Graph shows individual % change in released glutamate concentration in embryos treated with ionomycin in the absence or presence of 100 nM tetanus toxin compared with untreated (100%), *N* = 6, *p < 0.05, 2-tail paired *t* test.

To determine whether expression of VGluT1 is functional and necessary for glutamate release, we performed live imaging of intact *X. laevis* embryos expressing a genetically encoded sensor of released glutamate, iGluSnFR (123D5-spacer-modified; L.T.'s lab). We confirmed that the sensor reports levels of extracellular glutamate concentration by adding glutamate to the bathing solution, which results in an increase in iGluSnFR signal (Fig. 2*A*). Results show that the sensor reports a stronger signal in the neuroectoderm compared with the non-neural ectoderm, suggesting higher glutamate release in the neural plate (Fig. 2*B*). In contrast, VGluT1 knockdown decreases iGluSnFR signal but not membrane-mCherry fluorescence in the affected neural plate (Fig. 2*C*). This decrease is not due to reduced expression of iGluSnFR because staining for level of expressed reporter (myc-tagged) reveals that expression is comparable in wild type and knockdown halves of neural plate (Fig. 2*D*). These results indicate that VGluT1 is expressed and necessary for glutamate release in neural plate stage embryos.

To further confirm release of glutamate in neural plate stage embryos and determine whether it is dependent on Ca^2+^ influx, as in synapses, we measured in whole live embryos glutamate release in the presence or absence of ionomycin, a Ca^2+^ ionophore, using iGluSnFR (Fig. 2*E*) or a fluorometric ELISA assay (Fig. 2*F*). Results show that Ca^2+^ influx increases glutamate release from neural plate stage embryos (Fig. 2*E*,*F*), while knocking down VGluT1 expression (Fig. 2*G*) or blocking vesicular neurotransmitter release by preincubating neural plate stage embryos with tetanus toxin (Fig. 2*H*) inhibits ionomycin-induced glutamate release (Fig. 2*G*,*H*). This indicates that mechanisms of Ca^2+^-mediated vesicular glutamate release are present in neural plate stage embryos.

### VGluT1 is necessary for Ca^2+^ dynamics and signaling in neural plate cells

**Figure 3.**
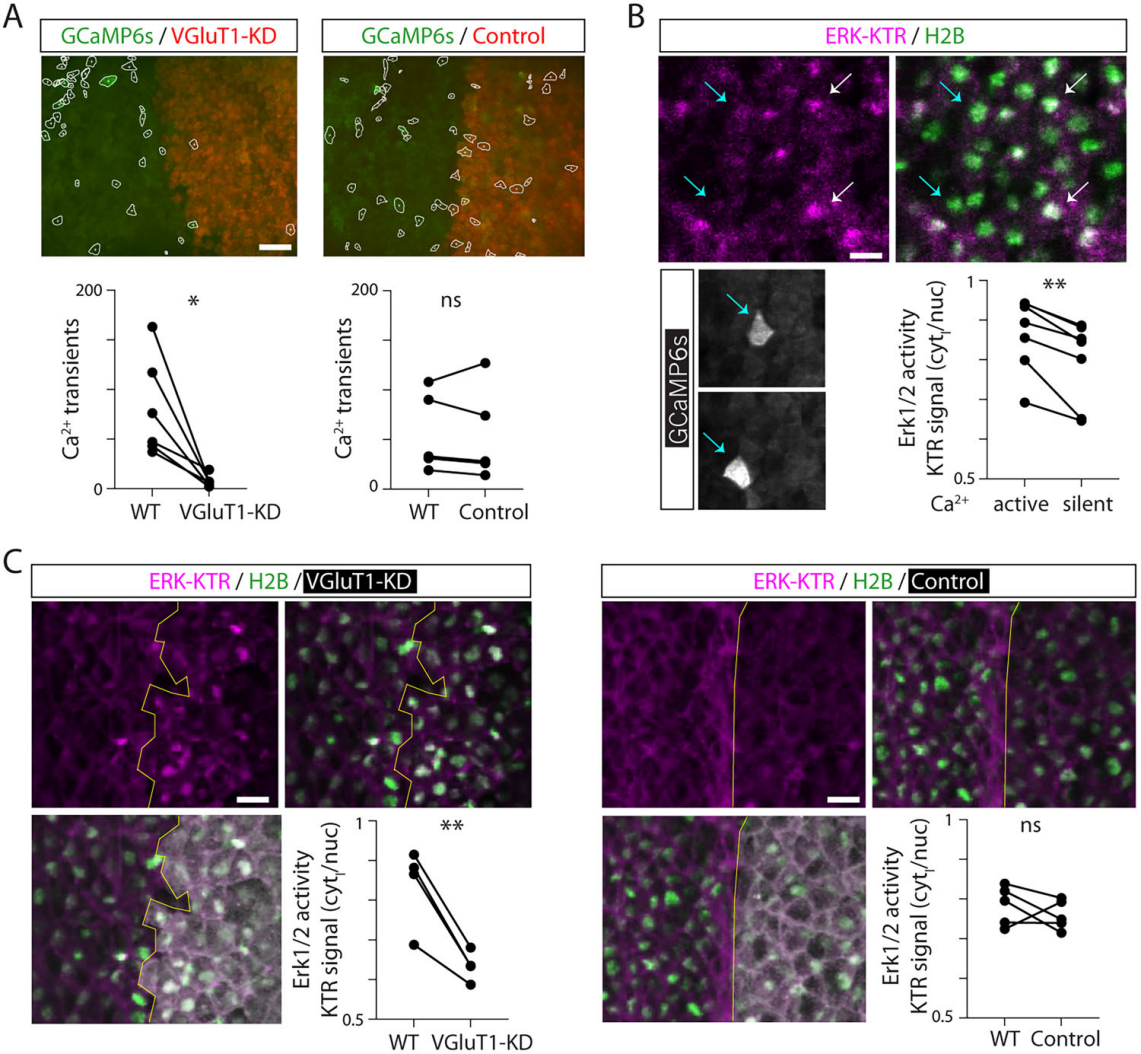
Ca^2+^ dynamics in the neural plate during folding depend on VGluT1 and activate Erk1/2. ***A***, Two-cell stage embryos were unilaterally VGluT1 knocked down (KD) and bilaterally injected with mRNA encoding GCaMP6s, Ca^2+^ reporter. Neural plate stage embryos were time-lapse imaged for 5 min and number of Ca^2+^ transients were measured. Image shows representative example of unilateral (red) VGluT1-KD and Control embryos. Circled cells are those exhibiting Ca^2+^ transients during recording. Graphs show individual number of Ca^2+^ transients per 5 min in WT and VGluT1-KD or Control halves of the neural plate. *N* = 6 VGluT1-KD and 5 Control embryos, *p < 0.05, ns, not significant, 2-tail paired *t* test. ***B***, Two-cell stage embryos were bilaterally injected with ERK-KTR-iRFP670 (Erk1/2 activity reporter), H2B-RFP (nuclear marker), and GCaMP6s. Neural-plate stage embryos were time-lapse imaged for 2 h, and cells exhibiting Ca^2+^ transients and those matching contralaterally their mediolateral and anteroposterior locations were selected for measurement of the ERK-KTR nuclear (delimited by H2B signal) and non-nuclear (cytosolic, delineated by GCaMP6 signal) fluorescence signal. ERK-KTR reports on active and inactive Erk1/2 with low and high nuclear versus cytosolic fluorescence intensity, respectively (Fig. S1). Top images are a representative example of single time frame of an embryo live imaged. Cyan arrows point to two cells exhibiting Ca^2+^ transients (active), and white arrows point to similarly positioned (mediolaterally and anteroposteriorly) cells that did not exhibit Ca^2+^ transients (silent) during recording. Bottom images show the two cells exhibiting Ca^2+^ transients during peak of transient. Graph shows mean ratio of ERK-KTR cytosolic/nuclear fluorescence intensity per embryo in active and silent cells. *N* = 7 embryos, *n* = 10 cells per embryo, **p < 0.01, 2-tail paired *t* test. ***C***, Two-cell stage embryos were bilaterally injected with ERK-KTR-iRFP670 and H2B-RFP and unilaterally with VGluT1-KD or Control along with tracer. Neural plate stage embryos were time-lapse imaged and ERK-KTR nuclear (delimited by H2B signal) and cytosolic fluorescence signal was measured in WT and injected neural plate cells. Images show single time frame of unilateral VGluT1-KD and Control embryos. Yellow line separates WT from injected half neural plate. Graph shows mean ratio of ERK-KTR cytosolic/nuclear fluorescence intensity in each half of the neural plate per embryo. *N* = 4 embryos, *n* = 10 cells per embryo (VGluT1-KD) and *N* = 5 embryos, *n* = 10 cells per embryo (Control), **p < 0.01, ns, not significant, two-tail paired *t* test. Scale bars, 20 μm.

Neural plate cells exhibit Ca^2+^ transients (Christodoulou and Skourides, 2015; Suzuki et al., 2017; Sequerra et al., 2018) that increase in frequency with the progression of neural plate folding (Sequerra et al., 2018) and are important for the morphogenesis of the neural tube (Christodoulou and Skourides, 2015; Suzuki et al., 2017). The increase in Ca^2+^ transient frequency in the neural plate correlates with progressive increase in the number of VGluT1-expressing neural plate cells (Fig. 1*C*). Hence, we assessed whether VGluT1 regulates neural plate cell Ca^2+^ dynamics. We find that knocking down VGluT1 expression in half of the embryo almost completely abolishes Ca^2+^ transients compared with the counterpart wild type neural plate (Fig. 3*A*), demonstrating a dependence on VGluT1-enabled glutamate signaling for Ca^2+^ dynamics in the neural plate.

Glutamate-mediated signaling is known to recruit various Ca^2+^-regulated pathways, including the MAPK cascade (Sweatt, 2004; Thomas and Huganir, 2004). Our previous study demonstrated that NMDA receptor activation elicits Erk1/2 phosphorylation in neural plate cells (Sequerra et al., 2018). We examined the status of Erk1/2 activity in neural plate cells by simultaneously live imaging Erk1/2 and Ca^2+^ activity in intact, neurulating embryos expressing specific, genetically encoded reporters. We first determined that the Erk1/2 reporter (ERK-KTR; Regot et al., 2014) expressed in *X. laevis* embryonic spinal cord and muscle cells reports on changes in Erk1/2 activity elicited by an agonist or antagonist of the pathway (Fig. S1). ERK-KTR is shuttled out of the nucleus when Erk1/2 is activated (Regot et al., 2014). Thus, higher Erk1/2 activity manifests as lower nuclear ERK-KTR signal intensity, while higher nuclear ERK-KTR signal intensity demonstrates inactive Erk1/2 (Fig. S1; Regot et al., 2014). We find that cells with Ca^2+^ transients (Ca^2+^ active) exhibit higher levels of Erk1/2 activity than those cells without Ca^2+^ transients (Ca^2+^ silent) during the recording (Fig. 3*B*). Importantly, VGluT1-deficient neural plate cells exhibit decreased Erk1/2 activity compared with the wild-type counterparts (Fig. 3*C*), suggesting that VGluT1-mediated glutamate release in the neural plate activates Erk1/2.

### VGluT1 is necessary for neural tube formation and regulates neural plate cell proliferation

**Figure 4.**
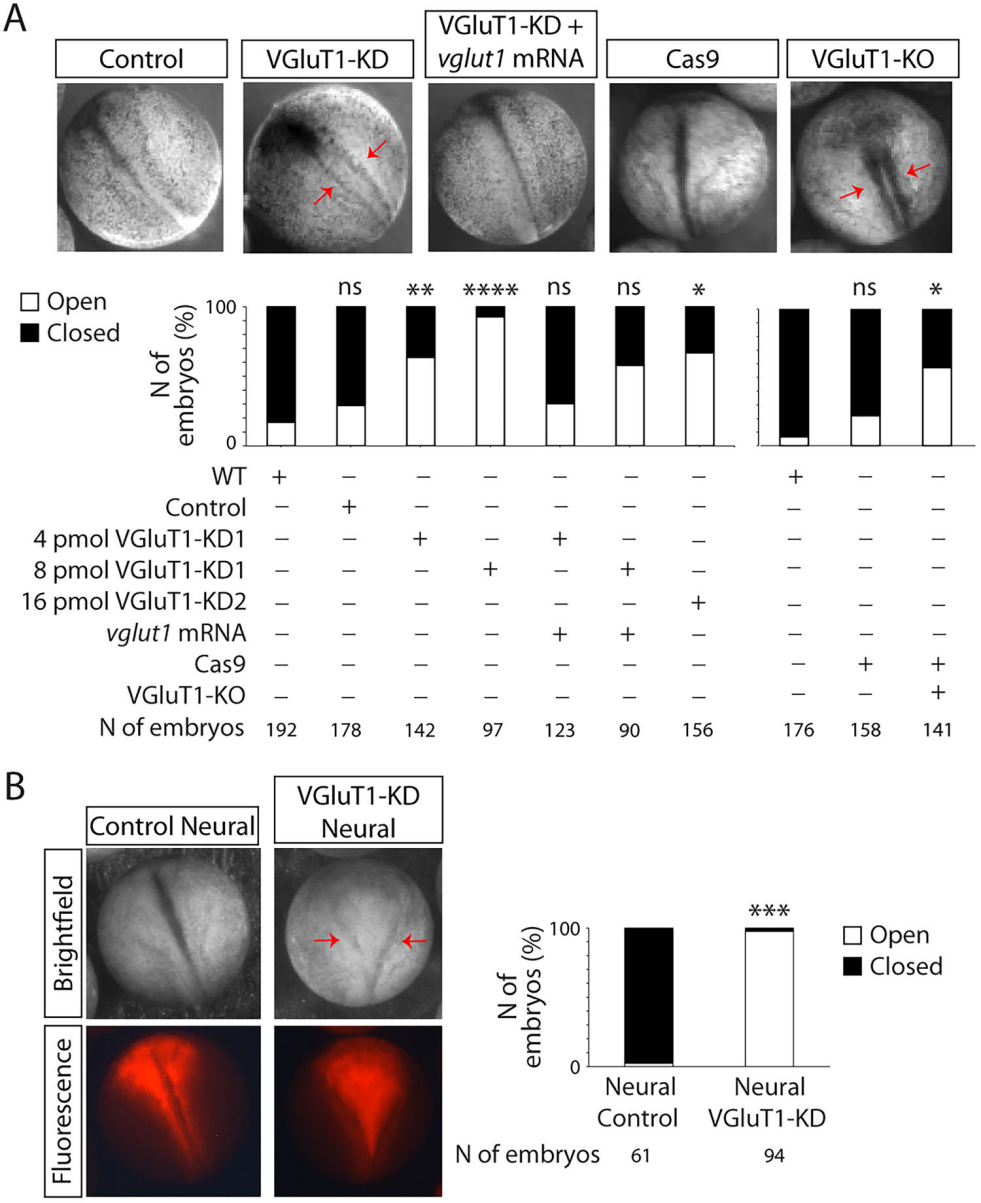
VGluT1 is necessary for neural tube formation. Two-cell (***A***) or 8-cell (***B***) stage embryos were bilaterally (***A***, ***B***) and dorsal blastomeres only (***B***) injected with 2–8 (***A***) or 2 (***B***) pmol VGluT1-morpholino 1 translation-blocking (VGluT1-KD1), 8 pmol VGluT1-morpholino 2 splicing-blocking (VGluT1-KD2, ***A***) or 8 (***A***) or 2 (***B***) pmol control-morpholino (Control) without (***A***, ***B***) or with (***A***) morpholino-resistant *vglut1* mRNA, or with Cas9 or Cas9 + VGluT1 sgRNA (VGluT1-KO, ***A***). Images (***A***, bright-field images in ***B***) show representative examples of embryos at the time neural tube closed in Control group. Red arrows indicate open neural tube. Fluorescence images in ***B*** indicate neural tissue-specific targeting of Control and VGluT1-KD. Graphs show % of embryos with open and closed neural tubes in each group. *N* ≥ 5 (***A***) and *N* = 3 (***B***) experiments, *n* of embryos indicated in graph for each group, *p < 0.05, **p < 0.01, ***p < 0.001, ****p < 0.0001, ns, not significant, one-way ANOVA, mixed-effects analysis with Geisser–Greenhouse correction, Dunnett's multiple-comparisons test, compared with WT (***A***) or two-tail paired *t* test (***B***).

The data thus far demonstrates that VGluT1 mediates glutamate release in the folding neural plate that is necessary for recruiting Ca^2+^ signaling during neural tube formation. We therefore investigated the importance of VGluT1-dependent glutamate release in neural tube closure. By knocking down (Fig. 1*B*) or knocking out (via CRISPR/Cas9) VGluT1 expression (Fig. S2) in developing embryos we find that VGluT1 deficiency leads to NTDs in *X. laevis* embryos that manifest as failure of closure of the neural tube in the midline (Fig. 4). The penetrance of VGluT1 knockdown-induced NTD phenotype correlates with the extent of knockdown (Fig. 1*E*) and is rescued by restoring VGluT1 expression (Fig. 4*A*), indicating specificity of VGluT1-KD-elicited NTD phenotype. In agreement with neuroectoderm-specific VGluT1 expression (Fig. 1*F,H*), knocking down VGluT1 only in neural tissue leads to NTDs (Fig. 4*B*).

**Figure 5.**
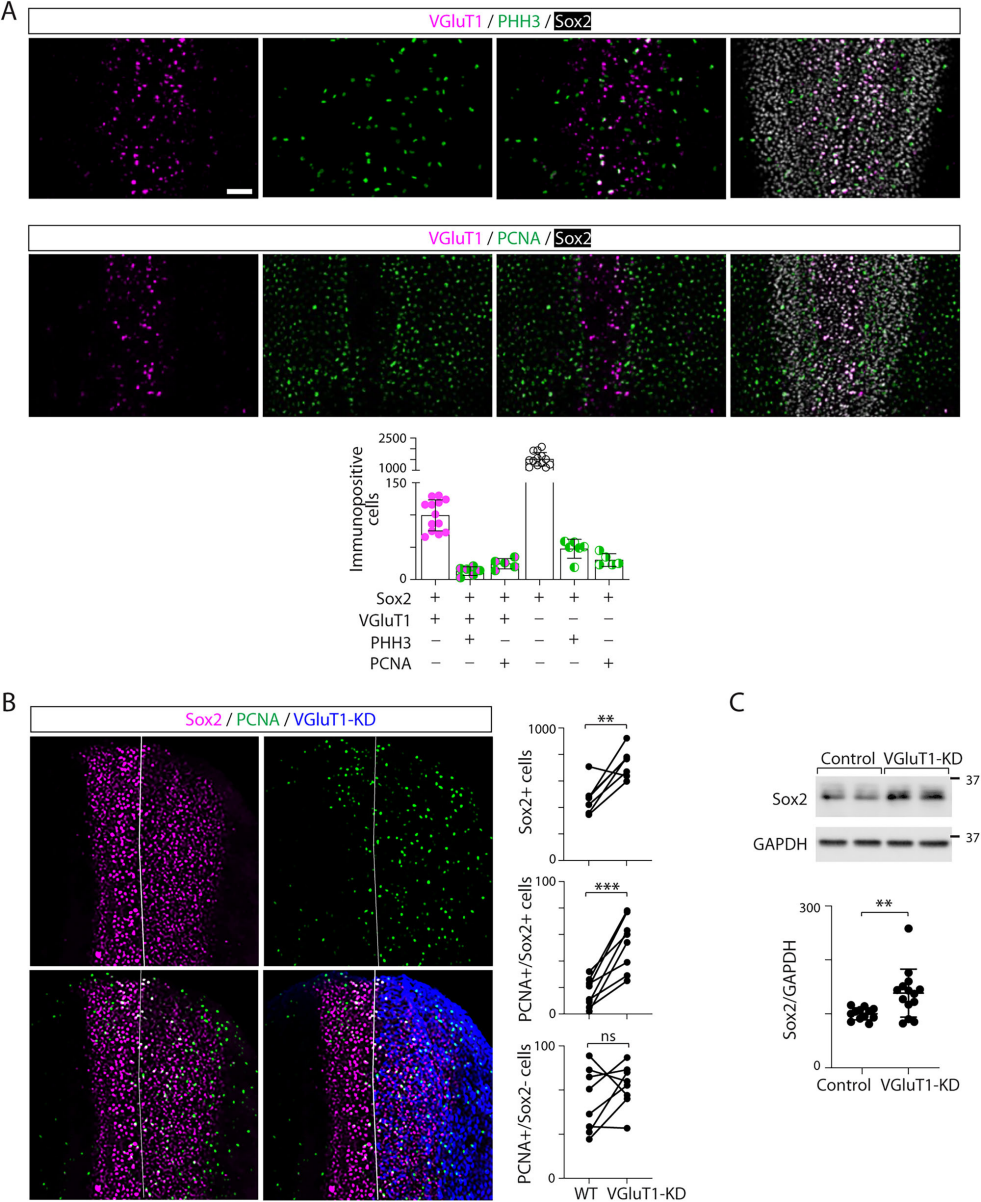
VGluT1 regulates neural plate cell proliferation and Sox2 expression. ***A***, Wild-type neural plate stage (st. 18) embryos were processed for whole-mount immunostaining for VGluT1, Sox2, and cell proliferation markers PCNA and PHH3. Shown are representative images. Graph shows individual and mean ± SD number of total and proliferation marker immunopositive VGluT1+ and VGluT1− neural plate cells (Sox2+). ***B***, Two-cell stage embryos were unilaterally injected with VGluT1-KD along with tracer. Neural plate stage (st. 18) embryos were processed for immunostaining. Images show representative example. Graphs show individual number of immunopositive (+) or immunonegative (−) cells in both halves of the neural plate per embryo. *N* = 7 embryos, **p < 0.01, ***p < 0.001, ns, not significant, 2-tail paired *t* test. ***C***, Two-cell stage embryos were bilaterally injected with VGluT1-KD or Control. Neural plate stage embryos were processed for Western blot assays for Sox2 and GAPDH, as loading control. Image is a representative example. Graph shows individual samples and mean ± SD normalized Sox2 levels. *N* = 5 experiments, **p < 0.01, 2-tail *t* test.

We find that most VGluT1-expressing cells are nonproliferative as revealed by the small proportion of VGluT1/PCNA or VGluT1/PHH3 coimmunopositive neural plate cells during neural plate folding (Fig. 5*A*). Moreover, overall, a small number of neural plate cells is proliferative in neural plate stage embryos (Fig. 5*A*). Hence, we examined whether VGluT1 is necessary for regulating neural plate cell proliferation during neural tube formation. Results show that VGluT1 knockdown increases the number of proliferative cells in the neural plate, evidenced by higher number of total Sox2+ and PCNA+/Sox2+ cells, while there is no significant difference in the number of proliferative cells that do not express Sox2 (PCNA+/Sox2−) (Fig. 5*B*). Western blot assays further reveal that VGluT1 knockdown increases Sox2 expression levels (Fig. 5*C*).

Altogether, these results indicate that VGluT1 regulates neural plate cell proliferation and enables neural tube morphogenesis.

## Discussion

The vesicular release of neurotransmitter has been primarily studied in the context of chemical synapses between neurons and target cells. Nevertheless, neurotransmitters and their synthetic and release machineries are known to be expressed prior to synapse formation (Lauder et al., 1981; Manent et al., 2005; Root et al., 2008). It could be argued that this precedent is needed for preassembling the specialized molecular platform required in synapses. Alternatively, it could indicate that neurotransmitter signaling plays a critical role even at early stages of nervous system development like the neural plate period. This study demonstrates that the vesicular release of glutamate in the neural plate occurs through mechanisms in part shared by those in synapses and that this release is indispensable for the formation of the neural tube. These findings attest that expression of glutamate release machinery is not merely preparatory for the synaptogenic period occurring later in development but that the signaling it elicits is essential for regulating neural cell proliferation during neural plate folding.

Here we show expression of components of the vesicular, Ca^2+^-regulated synaptosomal associated protein receptor (SNARE) complex machinery and VGluT1 immunogold particles localizing to vesicular ultrastructure in the proximity of the apicolateral membrane of neural plate cells. Moreover, we show that glutamate release is VGluT1 dependent and enhanced by Ca^2+^ influx. This suggests that the SNARE-dependent vesicular mechanism is present in neural plate stages. The extent to which this mechanism of vesicular glutamate release resembles the quantal release seen in synapses requires further investigation. Nevertheless, it appears that expression of vesicular release machinery and ultrastructure characteristic of dynamic exocytic and endocytic events, indicative of vesicular release, are staples of the folding neural plate across vertebrates (Portch and Barson, 1974; Takeuchi and Takeuchi, 1980; Shepard et al., 1998; Ybot-Gonzalez and Copp, 1999; Lee and Harland, 2010; Kim and Han, 2011; Ossipova et al., 2014). The morphogenesis of other tissues is also dependent on exocytosis, including the dorsal mesoderm in *X. laevis* (Kreis et al., 2022) and the *Drosophila* air sac primordium (Huang et al., 2019), where vesicular release of glutamate through SNARE-dependent contacts between cytonemes protruding from disc cells and air sac primordium cells has been reported (Huang et al., 2019). Therefore, specialized vesicular glutamate release might be a key mechanism that enables embryonic morphogenesis.

Vesicular release is a more energetically demanding process than alternative, nonquantal release mechanisms but offers more spatiotemporal regulation and organization of the signaling. The presence of this mechanism in neural plate stages suggests that this precision in signaling enabled by the vesicular release of glutamate is important for the specific cellular processes taking place during neural plate folding. Indeed, we find that Ca^2+^ transients, which are strongly dependent on VGluT1-mediated glutamate release, initially occur in individual neural plate cells, with clusters of coactive cells only becoming apparent later in the folding process (Christodoulou and Skourides, 2015; Suzuki et al., 2017; Sequerra et al., 2018). This suggests that the signaling is initially targeted to individual cells during the onset of neural plate folding, allowing for cell autonomy by restricting the release of glutamate to specific cells and subcellular domains.

**Figure 6.**
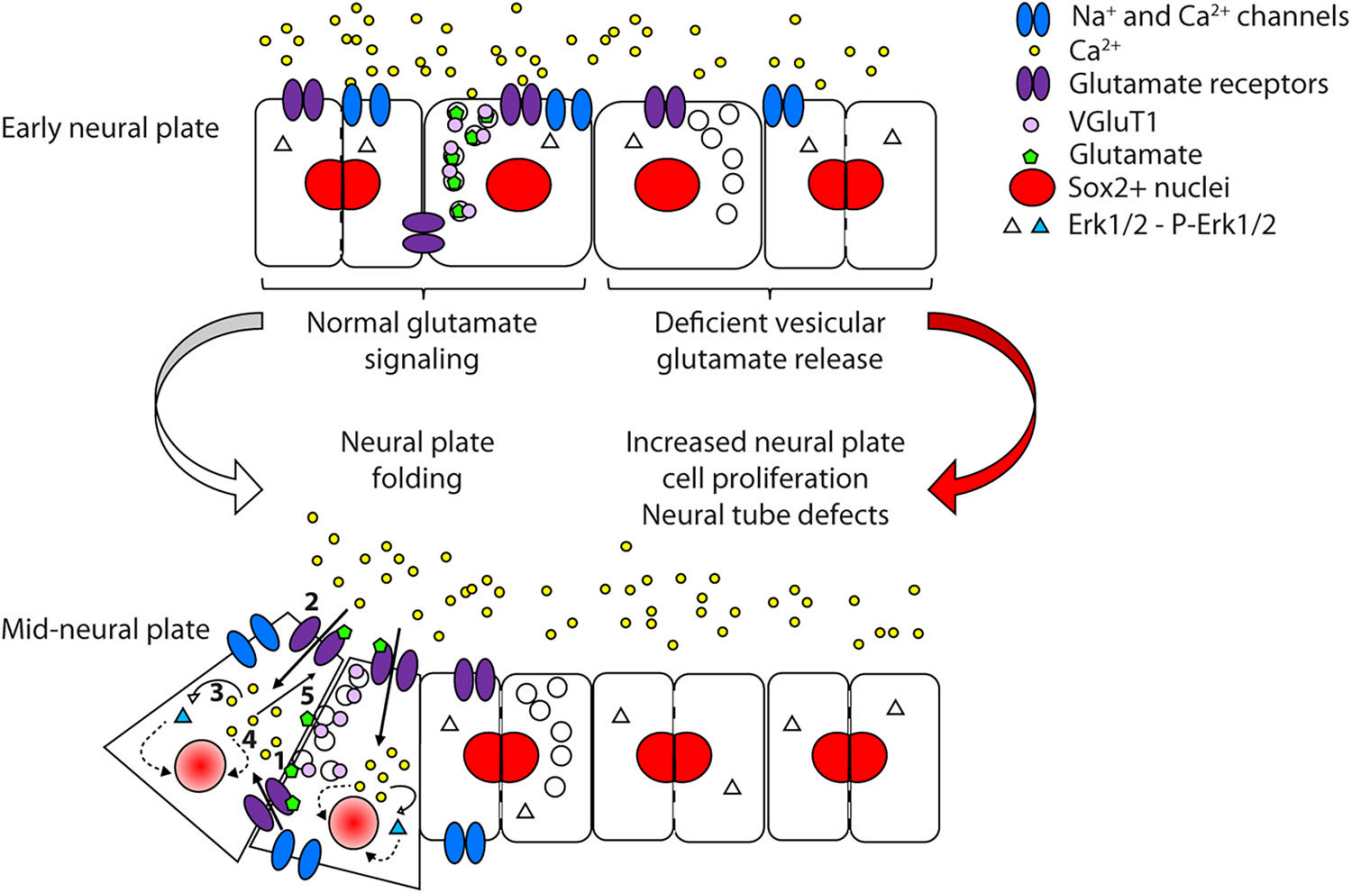
Model of mechanism of VGluT1-dependent regulation of neural tube formation. Vesicular release of glutamate (1) activates glutamate receptors eliciting Ca^2+^ transients in neural plate cells (2), which are necessary for the regulation of neural plate cell proliferation by downregulating Sox2 expression potentially (dashed arrows) through the recruitment of Erk1/2 (3) and/or other signaling pathways (4). Ca^2+^ transients are also necessary for the changes in cell shape (5) required for the timely folding of the neural plate during neural tube morphogenesis.

The locally released glutamate and subsequent Ca^2+^ dynamics in individual cells may be necessary for cell-autonomously regulating the cellular behaviors necessary for their participation in the folding of the neural plate. These include decisions on whether a cell should exit the cell cycle to change shape and migrate or continue proliferating to achieve the necessary cell count for neural tube morphogenesis. The VGluT1-dependent Ca^2+^-Erk1/2 activity that neural plate cells exhibit, demonstrated here, together with previous findings showing that the NTD phenotype from NMDA receptor knockdown is rescued by expressing an inducible, constitutively active MAPK during neural tube formation (Sequerra et al., 2018), suggest that Erk1/2 is downstream glutamate-dependent Ca^2+^ signaling in the regulation of neural plate cell proliferation necessary for neural tube morphogenesis (Fig. 6). Nevertheless, further investigation is needed to determine the full downstream signaling and cellular mechanisms of glutamate signaling.

Sox2 is a crucial transcription factor for the maintenance of neural cell stemness (Pevny and Rao, 2003) by supporting neural stem cell self-renewal in the developing and adult nervous system (Graham et al., 2003; Suh et al., 2007). Its expression is tightly regulated and alterations in this regulation have profound consequences, with constitutive expression inhibiting neuronal differentiation and downregulation leading to premature cell cycle exit and neurogenesis (Graham et al., 2003). We demonstrate that proper Sox2 levels in neural plate cells are dependent on VGluT1 expression which indicates that released glutamate regulates Sox2 expression. This finding reveals a mechanism by which glutamate signaling regulates neural plate cell proliferation. Given that Sox2 expression is negatively correlated to the frequency of Ca^2+^ transients during neural plate folding (Sequerra et al., 2018), it suggests that Ca^2+^ signaling could regulate Sox2 levels transcriptionally, as previously shown for Ca^2+^-dependent regulation of Sox2 expression in *Xenopus* developing neural tube (Shim et al., 2023) and/or posttranslationally, through phosphorylation-dependent mechanisms (Lim et al., 2017). We propose a model where VGluT1-dependent, Ca^2+^-mediated release of glutamate in selected neural plate cells signals the need for cells to withdraw from the cell cycle by regulating Sox2 expression, thereby enabling cell shape changes crucial for neural tube morphogenesis (Fig. 6).

This study underscores the critical role of neural activity and signaling during neural tube formation. Thus, the neural plate and the formation of the neural tube are vulnerable to exposure to environmental factors that can disrupt this neural signaling. Our study provides a foundational understanding of the mechanisms involved in neural tube morphogenesis, which can serve as a mechanistic basis to further investigate how neuroactive drugs interfere with this process, thereby advocating for the cautious use of such therapeutics during pregnancy.
